# Depression, quality of life, activities of daily living, and subjective memory after deep brain stimulation in Parkinson disease—A reliable change index analysis

**DOI:** 10.1002/gps.5184

**Published:** 2019-08-06

**Authors:** Gisela Pusswald, Patrick Wiesbauer, Walter Pirker, Klaus Novak, Thomas Foki, Johann Lehrner

**Affiliations:** ^1^ Department of Neurology Medical University of Vienna Vienna Austria; ^2^ Department of Neurology Wilhelminenspital Vienna Austria; ^3^ Department of Neurosurgery Medical University of Vienna Vienna Austria; ^4^ Department of Neurology Universitätsklinik Tulln Tulln Austria

**Keywords:** deep brain stimulation (DBS), Parkinson disease (PD), psychosocial outcome, reliable change index, subthalamic nucleus (STN)

## Abstract

**Objectives:**

In the field of Parkinson disease (PD) research, many studies have shown that deep brain stimulation (DBS) can soften side effects, which arise during long‐term medical therapy. This study focuses on the changes in depressive symptoms, quality of life (with the subdivisions physical and mental health), activities of daily living, and subjective memory functioning in PD patients testing the baseline and the outcome 1 year after DBS.

**Methods:**

For the first time, the reliable change index (RCI) methodology was applied to compare PD‐DBS patients (n = 22) with best medically treated PD patients (PD‐BMT; n = 28), subjects with mild cognitive impairment (MCI, n = 43) and healthy controls (n = 25) in the above‐mentioned domains. The used questionnaires included the revised Beck Depression Inventory (BDI‐II), the Short Form (36) Health Survey (SF‐36), the Bayer Activities of Daily Living Scale (B‐ADL), and the Forgetfulness Assessment Inventory (FAI).

**Results:**

The reliable change indices show high constant or improved results of the PD‐DBS patients in the domains subjective memory (85.7%‐100.0%), activities of daily living (60.0%‐90.0%), physical health summary (77.8%), depressive symptoms (61.9%), and mental health summary (50.0%) in comparison with the PD‐BMT, MCI, and control group.

**Conclusions:**

DBS is an established alternative to best medical treatment of PD. The comparisons between the PD‐DBS and PD‐BMT groups do suggest that the domains mental health, depressive symptoms, and physical health benefit most, while the domains activities of daily living and subjective memory functioning are rather constant. Nevertheless, further research is needed to identify mechanisms and predictors that lead to improvement in individual cases.

**Key points**

- This study focuses on changes in depressive symptoms, quality of life (with the subdivisions physical and mental health), activities of daily living, and subjective memory functioning in PD 1 year after DBS.
- We compare PD‐DBS patients with best medically treated PD patients, subjects with mild cognitive impairments and healthy controls.
- For comparison, we used the reliable change index (RCI) methodology.
- The comparisons between the PD‐DBS and PD‐BMT groups do suggest that the domains mental health, depressive symptoms, and physical health benefit most, while the domains activities of daily living and subjective memory functioning are rather constant.

## 1 INTRODUCTION

Deep brain stimulation (DBS) of the subthalamic nucleus is an established treatment for severe motor complications in Parkinson disease (PD), and since it is usually a lifelong therapy, it is essential to carefully evaluate beneficial and inadvertent effects in the long term. Studies demonstrate a remarkable improvement of motor symptoms in PD patients, whereas psychosocial impacts of DBS surgery including social adjustment, coping strategies, and mental health–related quality of life may be variable.^1^, ^2^, ^3^, ^4^ It is particularly difficult to determine whether the postoperative nonmotor deficiencies are related to the progression of the disease itself, to surgery or permanent stimulation.

The gold standard to evaluate the nonmotor symptoms in PD would be a randomized controlled trial, but this is not suitable for assessing single patients in the clinical setting—therefore, the reliable change index (RCI) analysis has been designed.^5^ The RCI analysis is capable of examining the influence of the disease progression over time and measures the real change in an individual case. Additionally, the standardized effect size according to the classification of Cohen has been calculated to assess the practical relevance of the changes. Recent studies have used this RCI methodology to assess the cognitive changes of deep brain stimulated Parkinson patients,^6^, ^7^, ^8^, ^9^, ^10^, ^11^ but so far, no study has used it on measuring the psychosocial outcome.

The purpose of this study was to test the hypotheses that there are no differences in the change of the domains depressive symptoms, health‐related quality of life, activities of daily living, and subjective memory functioning between the outcome of single PD‐DBS patients compared with the PD‐BMT, mild cognitive impairment (MCI), and control group. Another important aim was to test practice effects of the DBS surgery on nonmotor symptom changes despite the progression of the disease itself by comparing the PD‐DBS with the PD‐BMT group as well as controlling for neurodegenerative effects by comparing the PD‐DBS with the MCI group and aging effects by comparing the PD‐DBS patients with a healthy control.

## 2 METHODS

### 2.1 Study design

The current data are part of a larger research project, the Vienna Mild Cognitive Impairment and Cognitive Decline in Parkinson Disease Study (VMCI‐CD‐PD Study). The VMCI‐CD‐PD Study is a prospective cohort study including consecutive, community‐dwelling PD patients who attend the movement disorder clinic for assessment of their Parkinsonism. The study protocol was in accordance with the Helsinki Declaration and approved by the Ethical Committee of the Medical University of Vienna.

### 2.2 Data collection

The study collective (n = 118) was divided into four groups. Two groups consisted of PD patients—one group received best medical treatment (PD‐BMT; n = 28) and the other received DBS and best medical treatment (PD‐DBS; n = 22). PD patients with motor symptom duration of at least 5 years, good response to levodopa and/or apomorphine, and drug resistant motor complications or drug resistant tremor were included. Patients suffering from secondary parkinsonian syndromes, atypical Parkinsonism, and patients with severe cognitive impairments, such as dementia and uncontrolled mental disorders, were excluded. In addition, subjects with comorbidities and structural brain lesions that precluded DBS surgery had to be excluded from the study. The evaluation was performed during the “on‐state.”

One group included patients with MCI (n = 43) defined according to the Petersen criteria. These patients complain about a defective cognition and show an abnormal cognitive function for their age but do not fulfill criteria for dementia. Activities of daily living are unimpaired.^12^ The healthy control group (n = 25) consisted of individuals without PD and without cognitive impairment.

Test‐retest interval was 12 months. The Mini‐Mental‐State Examination (MMSE)—to assess the cognitive state^13^—and the “Wortschatz‐Test” (WST‐IQ)—to asses verbal intelligence levels and speech comprehension^14^—were used as comparability parameters between the different groups. Furthermore, all study participants had to answer the following study relevant questionnaires:

Beck Depression Inventory (BDI‐II) is a 21‐item instrument to quantify the level of depression by asking how often the subject felt certain ways within the past 2 weeks rated on a 4‐point scale ranging from 0 (no symptoms) to 3 (very intense symptoms) with a maximum score of 63. The results categorize the severity of depressive symptoms in minimal (0‐13), mild (14‐19), moderate (20‐28), and severe (29‐63); scores above 10 indicate clinically relevant depressive symptoms.^15^, ^16^

Short Form (36) Health Survey (SF‐36) is a 36‐item questionnaire constructed to survey health‐related quality of life delivering an 8‐scale profile of functional health and well‐being scores as well as a physical and mental health summary, the latter two being used in the current study. The score ranges from 0 to 100—lower scores reflect worse subjective quality of life.^17^

Bayer Activities of Daily Living Scale (B‐ADL) is a 25‐item questionnaire used to assess instrumental (eg, shopping and food preparation) and noninstrumental (eg, managing everyday activities and finding the way) activities of daily living. Ratings on a 10‐point scale between “never” and “always” sum up to a total score between 0 and 10—higher scores reflect increased impairment of everyday activities.^18^

Forgetfulness Assessment Inventory (FAI) is a 16‐items questionnaire used to measure subjective memory complaints scored on the basis of a 5‐point scale between “never” and “very often.” For statistical analysis, the average score across all items was used—higher scores reflect poorer subjective memory functioning.^19^

All groups were comparable for age, education, WST‐IQ, and BDI‐II and did not show statistically significant differences for age, education, WST‐IQ, and BDI‐II. The MMSE score showed a significant group difference *P* = .001 (*η* ^2^ = .085) with a small to medium effect. The follow‐up analysis via linear contrasts revealed that the healthy control group had higher MMSE values compared with the other groups (*P*s ≤ .019). The difference for the patient groups was negligible. See Table 1 for details.

**Table 1:** Description of the patient base

Group	n	m/f	Age^a^	Education^a^	MMSE	WST‐IQ	BDI‐II
Control	25	8/17	64.44 (±5.48)	11.64 (±3.64)	29.24 (±.78)	110.33 (±11.31)	9.12 (±6.82)
MCI	43	24/19	65.17 (±6.84)	11.67 (±4.09)	28.30 (±1.28)	108.40 (±11.99)	10.19 (±6.16)
PD‐BMT	28	15/13	63.10 (±7.43)	10.57 (±2.64)	28.43 (±1.20)	103.93 (±9.49)	9.21 (±5.69)
PD‐DBS	22	10/12	59.69 (±10.28)	10.77 (±3.69)	28.36 (±1.50)	107.62 (±13.59)	9.77 (±6.91)
Total	118	57/61	63.50 (±7.66)	11.24 (±3.61)	28.54 (±1.26)	107.58 (±11.68)	9.65 (±6.28)
Significance			.148	.459	.001	.153	.889

Abbreviations: BDI‐II, Beck Depression Inventory; m, male; f, female; MCI, mild cognitive impairment; MMSE, Mini‐Mental State Examination; PD‐BMT, best medically treated Parkinson disease group; PD‐DBS, Parkinson disease patients with deep brain stimulation; WST‐IQ, vocabulary test.

a In years.

### 2.3 Statistical analyses

As PD is a progressive disorder, neuropsychological follow‐up measurements are particularly important in individual case diagnosis. A distinction has to be made between general and differential changes in cognition. Thus, cognitive decline is also possible with constant fine motor skills or unchanged affectivity. Even therapeutic measures can influence cognitive, affective, or psychosocial variables. Temporary changes due to medication, impaired motor functioning, depressive symptoms, short‐term fluctuations, or freezing need to be taken into consideration. In order to meet those demands, the retest reliability (*r*_*tt*_), which was, in this study, retrieved via correlation analysis, has to be known. As a broadly examined phenomenon in serial assessments in neuropsychological research, practice effects—due to natural recovery, intervention, or prior exposure to the questionnaires used—have to be acknowledged and minimized by using the adjusted RCI formula that controls it.^5^, ^20^, ^21^

(1) $$\mathrm{RCI}=\left(\left(X_{2}-X_{1}\right)-\left(M_{2}-M_{1}\right)\right)*\mathrm{SED}.$$

The difference (*X*_2_ − *X*_1_) describes the individual change in performance of a test person. (*M*_2_ − *M*_1_) reflects the practice effect on retesting of the respective group—PD‐BMT, MCI, or control. The standard error of difference (SED) is useful for the individual case diagnosis for elderly people, since it allows distinguishing between cognitive deterioration due to illness and general age‐related degradation of cognitive capacity.^5^, ^20^, ^21^, ^22^

(2) $$\mathrm{SED}=\sqrt{\left(2*{\mathrm{SEM}}^{2}\right)}.$$

(3) $$\mathrm{SEM}=\mathrm{SD}\sqrt{\left(1-r_{\mathrm{tt}}\right)}.$$

Ringendahl assumes that a change is significant for a *z* value of ±1.64 (*P* = .05, one‐tailed).^20^ So these values are used to calculate the confidence intervals for determining the upper and lower limits for significant changes. The results of the individual DBS test persons RCIs are then compared with the limits of the PD‐BMT, MCI, and control group.

(4) $$\mathrm{CI}=\pm 1.64*\mathrm{SED}+\left(M_{2}-M_{1}\right).$$

To assess the practical relevance of changes found by the RCI comparisons, the standardized effect size has been calculated using the pretest‐posttest‐control (PPC) design for Cohen *d*. This estimation uses the pretest and posttest mean values of the treatment group (*T*, referring to the PD‐DBS patients) and control group (*C*, referring to the PD‐BMT, MCI and healthy control) as well as a pooled pretest standard deviation (*SD*_*pre*_). Although the bias of this equation is quite small when the size of each group (n) is greater than 10, a bias adjustment (*c*_*P*_) is added to get an approximately unbiased result.^23^ The absolute values of Cohen *d* can be interpreted as follows:|*d*| ≥ 0.20 is a small,|*d*|≥ 0.50 is a medium, and|*d*|≥ 0.80 is a large effect.^24^

(5) $$d_{\mathrm{ppc}2}=c_{P}\left[\frac{\left(M_{\text{post},T}-M_{\mathrm{pre},T}\right)-\left(M_{\text{post},C}-M_{\mathrm{pre},C}\right)}{{\mathrm{SD}}_{\mathrm{pre}}}\right],$$

(6) $${\mathrm{SD}}_{\mathrm{pre}}=\sqrt{\frac{\left(n_{T}-1\right){\mathrm{SD}}_{\mathrm{pre},T}^{2}+\left(n_{C}-1\right){\mathrm{SD}}_{\mathrm{pre},C}^{2}}{n_{T}+n_{C}-2}},$$

(7) $$c_{P}=1-\frac{3}{4\left(n_{T}+n_{C}-2\right)-1}.$$

## 3 RESULTS

Based on the 95% confidence intervals for deterioration and improvement of the PD‐BMT, MCI, and healthy control group, comparisons with the individual RCI results of the PD‐DBS patients were made. Necessary factors to calculate the confidence intervals are presented in Tables 2, 3, 4—including raw values of all PD‐DBS patients as well as mean values and standard deviations for all groups. The results of the RCI analysis comparing the PD‐DBS patients with the calculated confidence intervals of the PD‐BMT, the MCI, and healthy control group can be seen in Table 5.

**Table 2:** Individual raw test and retest values for the PD‐DBS group

Subject ID	Depressive Symptoms (BDI‐II)			Physical Health Summary (SF‐36)			Mental Health Summary (SF‐36)			Activities of Daily Living (B‐ADL)			Subjective Memory Functioning (FAI)		
	*X*_1_	*X*_2_	*X*_2_ − *X*_1_	*X*_1_	*X*_2_	*X*_2_ − *X*_1_	*X*_1_	*X*_2_	*X*_2_ − *X*_1_	*X*_1_	*X*_2_	*X*_2_ − *X*_1_	*X*_1_	*X*_2_	*X*_2_ − *X*_1_
1	3.00	2.00	−1.00	26.90	47.84	20.94	62.38	56.50	−5.88	1.79	1.16	−0.63	1.31	2.07	0.76
2	14.00	19.00	5.00	21.61	24.36	2.75	53.98	42.10	−11.88	8.35	3.40	−4.95	3.27	2.87	−0.40
3	12.00	5.00	−7.00	‐	‐	‐	‐	‐	‐	‐	0.17	‐	3.25	0.00	−3.25
4	10.00	‐	‐	‐	‐	‐	‐	‐	‐	7.48	6.00	−1.48	3.06	3.75	0.69
5	7.00	10.00	3.00	23.36	18.62	−4.74	52.09	61.94	9.85	4.48	4.20	−0.28	3.38	2.94	−0.44
6	17.00	32.00	15.00	21.26	29.08	7.82	37.39	37.26	−0.13	6.00	4.64	−1.36	1.81	2.00	0.19
7	5.00	10.00	5.00	34.99	23.83	−11.16	44.98	58.67	13.69	4.16	3.63	−0.53	2.94	3.33	0.39
8	0.00	9.00	9.00	26.38	30.18	3.80	53.21	50.75	−2.46	3.60	3.04	−0.56	2.73	3.25	0.52
9	6.00	4.00	−2.00	30.67	23.89	−6.78	43.70	62.98	19.28	1.60	1.64	0.04	2.06	2.30	0.24
10	8.00	11.00	3.00	27.88	27.88	0.00	43.44	43.44	0.00	2.04	2.04	0.00	1.38	1.38	0.00
11	4.00	4.00	0.00	‐	38.33	‐	‐	60.05	‐	1.60	3.39	1.79	2.87	3.07	0.20
12	24.00	17.00	−7.00	24.27	28.98	4.71	41.39	48.33	6.94	4.92	3.84	−1.08	1.94	2.50	0.56
13	16.00	11.00	−5.00	31.08	39.22	8.14	39.48	49.54	10.06	‐	8.57	‐	‐	3.36	‐
14	4.00	3.00	−1.00	24.43	27.72	3.29	64.49	67.67	3.18	3.70	1.44	−2.26	1.88	2.19	0.31
15	24.00	14.00	−10.00	34.37	37.87	3.50	34.04	44.14	10.10	2.48	2.36	−0.12	2.93	3.75	0.82
16	6.00	4.00	−2.00	33.60	41.18	7.58	49.59	49.07	−0.52	1.00	1.68	0.68	2.00	0.00	−2.00
17	12.00	8.00	−4.00	32.34	45.73	13.39	47.07	56.83	9.76	3.00	1.80	−1.20	2.90	2.75	−0.15
18	20.00	4.00	−16.00	42.41	52.21	9.80	30.35	47.60	17.25	2.72	1.96	−0.76	2.06	2.31	0.25
19	5.00	9.00	4.00	43.15	48.88	5.73	56.50	34.96	−21.54	1.48	2.04	0.56	2.50	3.00	0.50
20	9.00	12.00	3.00	37.42	48.56	11.14	51.63	31.32	−20.31	3.16	4.92	1.76	3.56	2.94	−0.62
21	1.00	1.00	0.00	‐	51.97	‐	‐	58.63	‐	1.36	1.08	−0.28	2.94	2.25	−0.69
22	8.00	4.00	−4.00	44.70	47.96	3.26	56.66	56.05	−0.61	1.00	1.52	0.52	2.69	2.88	0.19

Abbreviations: B‐ADL, Bayer Activities of Daily Living Scale; BDI‐II, Beck Depression Inventory; FAI, Forgetfulness Assessment Inventory; SF‐36, Short Form (36) Health Survey; *X*_1_, individual test value; *X*_2_, individual retest value; *X*_2_ − *X*_1_, difference between retest and test value (gray‐shaded fields stand for individual improvement).

**Table 3:** Mean values and standard deviations of all study groups

Parameter		PD‐DBS			PD‐BMT			MCI			Control			n Total
		Mean	SD	n	Mean	SD	n	Mean	SD	n	Mean	SD	n	
BDI‐II	Depressive symptoms test	9.76	7.1	21	9.21	5.7	28	10.19	6.2	43	9.12	6.8	25	117
	Depressive symptoms retest	9.19	7.2		8.64	7.2		9.19	6.3		9.00	7.1		
SF‐36	Physical health summary test	31.16	7.4	18	41.63	8.2	23	46.16	10.2	37	48.53	7.6	20	98
	Physical health summary retest	35.78	10.9		38.97	11.2		44.24	10.6		48.79	7.0		
	Mental health summary test	47.91	9.4		52.36	6.3		49.90	10.5		48.69	9.2		
	Mental health summary retest	49.95	10.0		52.34	9.1		52.48	8.2		50.74	8.7		
B‐ADL	Activities of daily living test	2.20	2.1	20	1.99	1.1	28	1.91	.8	40	1.66	0.6	25	113
	Activities of daily living retest	2.79	1.4		2.45	1.8		2.25	1.6		1.61	0.5		
FAI	Subjective memory functioning test	2.55	0.7	21	2.50	0.7	27	3.12	0.6	42	2.70	0.6	24	114
	Subjective memory functioning retest	2.45	1.0		2.51	0.7		2.95	0.6		2.82	0.7		

Abbreviations: B‐ADL, Bayer Activities of Daily Living Scale; BDI‐II, Beck Depression Inventory; FAI, Forgetfulness Assessment Inventory; MCI, mild cognitive impairment group; PD‐BMT, best medically treated Parkinson disease group; PD‐DBS, Parkinson disease patients with deep brain stimulation; SD, standard deviation; SF‐36, Short Form (36) Health Survey.

**Table 4:** *r*_*tt*_, SEM, SED, and CI

Parameters for PD‐BMT	*r*_*tt*_	SEM	SED	SD	*M*_2_ − *M*_1_	CI−	CI+	n
Depressive Symptoms (BDI‐II)	.83	2.37	3.36	5.7	−0.57	−6.07	4.94	28
Physical Health Summary (SF‐36)	.81	3.57	5.04	8.2	−2.66	−10.93	5.61	23
Mental Health Summary (SF‐36)	.28	5.36	7.58	6.3	−0.02	−12.45	12.40	23
Activities of Daily Living (B‐ADL)	.27	0.98	1.39	1.1	0.47	−1.80	2.74	28
Subjective Memory Functioning (FAI)	.86	0.25	0.35	0.7	0.04	−0.61	0.54	27
Parameters for MCI								
Depressive Symptoms (BDI‐II)	.64	3.71	5.24	6.2	−1.00	−9.60	7.59	43
Physical Health Summary (SF‐36)	.60	6.42	9.08	10.2	−1.92	−16.81	12.97	37
Mental Health Summary (SF‐36)	.66	6.08	8.59	10.5	2.62	−11.47	16.72	37
Activities of Daily Living (B‐ADL)	.32	0.68	0.96	0.8	0.34	−1.23	1.91	40
Subjective Memory Functioning (FAI)	.57	0.40	0.56	0.6	−0.16	−1.08	0.76	42
Parameters for Control								
Depressive Symptoms (BDI‐II)	.83	2.79	3.94	6.8	−0.12	−6.58	6.34	25
Physical Health Summary (SF‐36)	.49	5.39	7.63	7.6	0.26	−12.25	12.77	20
Mental Health Summary (SF‐36)	.58	6.01	8.50	9.2	2.05	−11.88	15.98	20
Activities of Daily Living (B‐ADL)	.85	0.24	0.33	0.6	−0.05	−0.59	0.50	25
Subjective Memory Functioning (FAI)	.64	0.35	0.50	0.6	0.12	−0.69	0.94	24

Abbreviations: B‐ADL, Bayer Activities of Daily Living Scale; BDI‐II, Beck Depression Inventory; CI−/CI+, confidence intervals; FAI, Forgetfulness Assessment Inventory; MCI, mild cognitive impairment group; *M*_2_ − *M*_1_, difference between retest‐ and test‐mean‐value reflecting practice effects on retesting; PD‐BMT, best medically treated Parkinson disease group; *r*_*tt*_, retest reliability; SED, standard error of difference; SEM, standard error of measurement; SD, standard deviation; SF‐36, Short Form (36) Health Survey.

**Table 5:** Results of the reliable change index comparisons

Parameter	PD‐DBS vs. PD‐BMT				PD‐DBS vs. MCI				PD‐DBS vs. healthy control				n Total
	|*d*_*ppc*2_|	⇓	⇔	⇑	|*d*_*ppc*2_|	⇓	⇑	⇑	|*d*_*ppc*2_|	⇓	⇔	⇑	
Depressive Symptoms (BDI‐II)	.000	38.10 (n = 8)	19.05 (n = 4)	42.86 (n = 9)	.067	38.10 (n = 8)	19.05 (n = 4)	42.86 (n = 9)	.064	38.10 (n = 8)	19.05 (n = 4)	42.86 (n = 9)	21
Physical Health Summary (SF‐36)	.920	22.22 (n = 4)	33.33 (n = 6)	44.44 (n = 8)	.696	22.22 (n = 4)	38.89 (n = 7)	38.89 (n = 7)	.580	22.22 (n = 4)	38.89 (n = 7)	38.89 (n = 7)	18
Mental Health Summary (SF‐36)	.262	50.00 (n = 9)	.00 (n = 0)	50.00 (n = 9)	.057	50.00 (n = 9)	5.56 (n = 1)	44.44 (n = 8)	.000	50.00 (n = 9)	5.56 (n = 1)	44.44 (n = 8)	18
Activities of Daily Living (B‐ADL)	.603	10.00 (n = 2)	80.00 (n = 16)	10.00 (n = 2)	.613	10.00 (n = 2)	70.00 (n = 14)	20.00 (n = 4)	.313	40.00 (n = 8)	30.00 (n = 6)	30.00 (n = 6)	20
Subjective Memory Functioning (FAI)	.147	14.29 (n = 3)	76.19 (n = 16)	9.52 (n = 2)	.114	.00 (n = 0)	90.48 (n = 19)	9.52 (n = 2)	.346	.00 (n = 0)	90.48 (n = 19)	9.52 (n = 2)	21

Abbreviations: B‐ADL, Bayer Activities of Daily Living Scale; BDI‐II, Beck Depression Inventory; *d*_*ppc*2_, effect size Cohen *d* pretest‐posttest design; FAI, Forgetfulness Assessment Inventory; MCI, mild cognitive impairment group; PD‐BMT, best medically treated Parkinson disease group; PD‐DBS, Parkinson disease patients with deep brain stimulation; SF‐36, Short Form (36) Health Survey; ⇓, deterioration in %; ⇔, no changes in %; ⇑, improvement in %.

### 3.1 PD‐DBS versus PD‐BMT

The RCI analysis showed an improvement of 42.86% and a deterioration of 38.10% of the PD‐DBS patients for depressive symptoms (BDI‐II) with no effect (|*d*_*ppc*2_| = .000). The SF‐36 physical health summary improved in 44.44% and deteriorated in 22.22% of the PD‐DBS patients with a large effect (|*d*_*ppc*2_| = .920). Equal improvements and deteriorations were found for the SF‐36 mental health summary—each 50.00% with a small effect (|*d*_*ppc*2_| = .262)—as well as the B‐ADL activities of daily living—each 10.00% with a medium effect (|*d*_*ppc*2_|= .603). Subjective memory functioning (FAI) did not change in 76.19%, worsened in 14.29%, and improved in 9.52% of the PD‐DBS patients with no effect (|*d*_*ppc*2_|= .147).

### 3.2 PD‐DBS versus MCI

Again, the depressive symptoms (BDI‐II) depicted an improvement of 42.86% and a deterioration of 38.10% of the PD‐DBS patients with no effect (|*d*_*ppc*2_| = .067). The SF‐36 physical health summary showed that 38.89% improved whereas 22.22% of the PD‐DBS patients deteriorated with a medium effect (|*d*_*ppc*2_| = .696). Mental health summary showed an improvement in 44.44% and deterioration in 50.00% of the PD‐DBS patients with no effect (|*d*_*ppc*2_| = .057). Activities of daily living (B‐ADL) showed no change in 70.00% with a medium effect (|*d*_*ppc*2_|= .613). Subjective memory functioning (FAI) portrayed no change in 90.48% of the PD‐DBS patients with no effect (|*d*_*ppc*2_|= .114).

### 3.3 PD‐DBS versus healthy control

The depressive symptoms (BDI‐II) showed an improvement of 42.86% and a deterioration of 38.10% of the PD‐DBS patients with no effect (|*d*_*ppc*2_| = .064). The SF‐36 physical health summary presented 38.89% improved and 22.22% of the PD‐DBS patients deteriorated with a medium effect (|*d*_*ppc*2_| = .580), while the mental health summary showed an improvement in 44.44% of patients and deterioration in 50.00% of the patients with no effect (|*d*_*ppc*2_| = .000). Activities of daily living (B‐ADL) depicted a deterioration of 40.00% and improvements in 30.00% of the patient with a small effect (|*d*_*ppc*2_|= .313). Subjective memory functioning (FAI) portrayed no change in 90.48% of the PD‐DBS patients with a small effect (|*d*_*ppc*2_|= .346).

Over all, the reliable change indices show high constant or improved results of the PD‐DBS patients in the domains subjective memory (85.7‐100.0%), activities of daily living (60.0‐90.0%), physical health summary (77.8%), depressive symptoms (61.9%), and mental health summary (50.0%) in comparison with the PD‐BMT, MCI, and control group.

## 4 DISCUSSION

The primary aim of this study was to assess the effect of DBS on depressive symptoms, physical and mental health‐related quality of life, activities of daily living, and subjective memory functioning. An RCI analysis was performed to compare the PD‐DBS patients with a PD‐BMT, an MCI, and a healthy control group. The results in all domains showed improved, constant, and worsened results. Overall, there was a change in individual patients, but the change was very heterogeneous with gains and losses.

Many studies cover the issues of depression in PD patients with and without PD‐DBS surgery, but none of them has used the BDI‐II questionnaire to enquire the severity of depression, nor have any of these studies used RCI analyses. Most of them do present conflicting results regarding the change in depressive symptoms in PD‐DBS patients, while recent meta‐analyses performed by Combs and Couto, however, verify the improvements also found in the current study.^25^, ^26^

The majority of the studies investigating quality of life demonstrated an improvement of the physical health—which is no surprise since DBS is known for its fast effects on the physical symptoms—and no change or even deterioration of the mental health summary due to the high expectations of the patients, the progression of PD itself, or side effects of the surgery.^1^, ^4^, ^27^, ^28^ As for the current study, the physical health summary showed improvements throughout all comparisons. The mental health summary improved in almost half of PD‐DBS patients when compared with the PD‐BTM, MCI, and healthy control group, while a deterioration of half of the PD‐DBS patients in all three comparisons could be demonstrated. These results indicate that further research is needed to be able to quantify the extent of possible mental side effects in specific patients.

The B‐ADL questionnaire was used in this study because of its applicability on patients with mild to moderate cognitive impairment. However, there are no other comparable studies using this score to assess activities of daily living in PD‐DBS patients. The PD‐DBS group showed no changes in comparison with the PD‐BMT and the MCI group, while compared with the healthy control, a slight deterioration was found. These results indicate that DBS has no adverse or improving effects on PD patients regarding activities of daily living. The results of the comparison between the PD‐DBS participants and the healthy control indicate that further research is needed to be able to quantify possible procedural side effects aside from possibly more impairment due to the nature of PD.

Subjective memory functioning (FAI) change of PD patients after neurosurgery has not been investigated so far. The current study revealed that the subjective memory functioning after PD‐DBS showed no change in all comparisons. Since over three quarter of the PD‐DBS patients achieved constant results, it is reasonable that PD‐DBS has no adverse effects on subjective memory.

Some limitations have to be taken into consideration when interpreting these findings. Unfortunately, not all PD‐DBS study participants answered all questionnaires, so there are some missing values, which had to be taken out of consideration (see Table 2). Due to the small sample size, only medium to large effects reached statistical significance. Natural regression effect is a limitation that might falsify the results by the means that the posttest measurements are shifted towards the middle of the distribution. Another limitation concerns the RCI—the fact that the test is taken twice does not necessarily guarantee that the variable is measured with the same precision. The elapsed time between the two tests can affect the reliability of scores across that period—shorter retest intervals lead to higher, longer intervals to lower reliability coefficients (*r*_*tt*_). The confidence interval characterizes the range of feature expressions in which 95% of the measured values are located and consequently represents another restriction for the RCI comparison, because the bigger the range of values is, the less precise the estimation becomes.^22^, ^24^, ^29^

## 5 CONCLUSION

In summary, DBS can be seen as an established alternative to the best medical treatment of PD. The results of the current study suggest that there are no general conclusions about the psychosocial improvement or deterioration that could be expected from the PD‐DBS surgery. However, it supports results of other studies regarding the enhancement of depressive symptoms and general physical quality of life aspects. Furthermore, it mostly proved no changes for activities of daily living (B‐ADL) and subjective memory (FAI).

In order to be able to estimate the psychosocial outcome after the deep brain stimulation individually for each patient as precisely as possible—despite the still existing uncertainties about the mechanisms and emerging impairments—further studies are necessary in order to find predictors for improvement or worsening and to develop optimal individual therapy options.

## CONFLICT OF INTEREST

None declared.

## AUTHOR CONTRIBUTIONS

Dr Foki collected the data and assisted discussing and writing the article. Dr Wiesbauer performed the statistical analysis and assisted discussing and writing the article. Dr Pirker collected the data and assisted with writing the article. Dr Novak collected the data and assisted with writing the article. Dr Pusswald collected the data and assisted with writing the article. Dr Lehrner designed the study, supervised the data collection, and wrote the paper.

## Data Availability

The data that support the findings of this study are available from the corresponding author upon reasonable request.
